# Investigating the relationship between cycle threshold of SARS-CoV-2 RT-PCR, clinical features, and laboratory data in hospitalized COVID-19 patients in Semnan, Iran

**DOI:** 10.3389/fcimb.2025.1522375

**Published:** 2025-03-24

**Authors:** Mohammad Hassan Kalantar Neyestanaki, Omid Gholizadeh, Saman Yasamineh, Mahdieh Tarahomi, Pegah Pooya, Majid Eslami, Mehdi Dadashpour, Hadi Ghaffari

**Affiliations:** ^1^ Department of Medicine, Arak University of Medical Science, Arak, Iran; ^2^ Research Center for Clinical Virology, Tehran University of Medical Sciences, Tehran, Iran; ^3^ Young Researchers and Elite Club, Islamic Azad University, Tabriz, Iran; ^4^ Student Research Committee, Semnan University of Medical Sciences, Semnan, Iran; ^5^ Department of Molecular Virology, Semnan Health Reference Laboratory, Semnan University of Medical Sciences, Semnan, Iran; ^6^ Cancer Research Center, Semnan University of Medical Sciences, Semnan, Iran; ^7^ Department of Bacteriology and Virology, Semnan University of Medical Sciences, Semnan, Iran

**Keywords:** COVID-19, RT-PCR, cycle threshold, laboratory data, Iran

## Abstract

**Introduction:**

COVID-19, caused by the SARS-CoV-2 virus, has emerged as a global public health crisis. Understanding the factors associated with disease severity and outcomes is crucial for effective patient management. This study aimed to investigate the association between cycle threshold (CT) values, demographic data, medical history, clinical manifestations, and laboratory findings in hospitalized COVID-19 patients in Semnan, Iran.

**Methods:**

A cross-sectional study was conducted on 86 patients with confirmed COVID-19 admitted to two hospitals in Semnan, Iran, between December 2022 and March 2023. Respiratory swab samples were collected RT-PCR was performed, CT values were obtained, and data were collected from medical records, including demographic information, medical history, clinical manifestations, and laboratory results. Statistical analysis was performed using SPSS software.

**Results:**

The study included 86 COVID-19 patients, with a slightly higher representation of females (55.8%) and a mean age of 67.43 years. Pre-existing conditions like hypertension, diabetes mellitus, and ischemic heart disease were prevalent among hospitalized patients. A majority of patients (59.3%) had severe COVID-19, as indicated by lower CT values, while 31.4% exhibited oxygen saturation levels below 90%. Significant differences were observed in FBS, CRP, WBC, Hb, Cr, and SPo2 levels between severe and non-severe patients. Correlation analysis revealed associations between age, CRP, Cr, BUN, FBS, Vitamin D, TG, LDL, HDL, AST, ALP, and SPo2. Reflecting complex interactions between inflammatory markers, organ function, and lipid metabolism in COVID-19 patients.

**Conclusion:**

This study provides valuable insights into the association between CT values, clinical characteristics, and laboratory findings in hospitalized COVID-19 patients. The findings underscore the importance of CT values in assessing disease severity and potential prognostication. Further research is warranted to validate these findings in larger and more diverse patient populations.

## 1 Introduction

In late December 2019, an outbreak of an unidentified respiratory infection emerged in Wuhan, China, quickly evolving into a global pandemic. On February 11, 2020, the World Health Organization (WHO) named the disease COVID-19 (Group, 2020; Hashimoto et al., 2022; Yasamineh et al., 2022). Since its emergence, COVID-19 has led to significant illness and loss of life, with more than 585 million confirmed cases and 6.4 million fatalities reported globally (Msemburi et al., 2022). In Iran, the earliest confirmed cases were detected in Qom on February 19, 2020, and within a matter of weeks, the virus had spread across the country due to its high level of transmissibility (Armin et al., 2022). SARS-CoV-2, the virus responsible for COVID-19, is an enveloped, positive-sense, single-stranded RNA virus that gains entry into human cells via its spike (S) protein binding to the angiotensin-converting enzyme 2 (ACE2) receptor. The S protein is composed of two subunits: S1, which facilitates receptor binding, and S2, which enables membrane fusion and viral entry. Once inside the host cell, the virus hijacks the host’s cellular machinery to replicate and produce new virions, promoting further infection (Hoffmann et al., 2020; Hu et al., 2021).

COVID-19 presents a broad spectrum of clinical manifestations, ranging from asymptomatic cases to severe respiratory distress and multi-organ failure. While most individuals experience mild symptoms, such as fever, cough, and fatigue, severe cases commonly involve dyspnea, hypoxemia, and complications like acute respiratory distress syndrome (ARDS), thromboembolism, and shock. Certain populations, including the elderly and individuals with preexisting conditions such as hypertension, diabetes, and cardiovascular diseases, are at higher risk of developing severe outcomes. Besides respiratory symptoms, the disease can also affect the gastrointestinal, neurological, and cardiovascular systems (Gavriatopoulou et al., 2020; Magadum and Kishore, 2020; Patel, 2020). Laboratory findings in COVID-19 patients often reveal lymphopenia, elevated inflammatory markers (including IL-6, TNF, and IL-10), and coagulation abnormalities. Markers such as increased neutrophil counts, C-reactive protein (CRP), and lactate dehydrogenase (LDH) have been linked to disease progression. Severe cases may also exhibit coagulopathy, leading to disseminated intravascular coagulation (DIC) and multi-organ failure, further complicating the prognosis (Liao et al., 2020; Lucas et al., 2020; Zhou et al., 2020).

Diagnostic methods for SARS-CoV-2 include molecular, serological, and radiological techniques. Real-time reverse transcription-polymerase chain reaction (RT-PCR) remains the gold standard due to its high sensitivity and specificity. The cycle Threshold (CT) value in RT-PCR testing serves as a surrogate marker of viral load; lower CT values indicate higher viral loads and are often associated with increased disease severity. However, interpreting CT values can be challenging as they are influenced by factors such as sample type, assay variability, and patient characteristics (Tahamtan and Ardebili, 2020; Teymouri et al., 2021). Recent studies have examined the relationship between CT values and various clinical parameters, including biochemical and hematological markers like LDH, lymphocyte counts, and troponin I. Correlations between CT values and inflammatory markers suggest that viral load may play a role in driving systemic inflammation and modulating the immune response. Despite these findings, comprehensive investigations that integrate CT values with demographic data, clinical symptoms, and laboratory results remain scarce, particularly in specific populations such as those in Iran (Singanayagam et al., 2020; Markewitz et al., 2024).

To address this gap in knowledge, the current study aims to explore the association between CT values, clinical features, and laboratory findings in hospitalized COVID-19 patients in Semnan, Iran. By analyzing the relationship between viral load and disease severity, this research seeks to provide valuable insights for risk assessment and clinical decision-making. Ultimately, these findings could inform better treatment strategies and improve patient outcomes.

## 2 Materials and methods

### 2.1 Study design and participants

This cross-sectional study was conducted on all individuals with respiratory symptoms who were referred to Kosar and Amir-Al-Momenin Hospitals of Semnan University of Medical Sciences between December 2022 and March 2023. The respiratory samples of these patients were sent to the Reference Health Virology Laboratory of the University for Identification of COVID-19 infection and had positive PCR results. Additionally, all their medical records and demographic information were registered in the hospital system. The sampling method was census and all complete patient files were included in the study.

A cross-sectional study was carried out on individuals with respiratory symptoms who were referred to Kosar and Amir-Al-Momenin Hospitals of Semnan University of Medical Sciences from December 2022 to March 2023. Respiratory samples collected from these patients were sent to the Reference Health Virology Laboratory of the University for molecular detection of COVID-19. The study included only those patients who tested positive via RT-PCR, and their medical records along with demographic details were obtained from the hospital database. The sampling method followed a census-based approach, incorporating all complete patient files.

### 2.2 Inclusion criteria

Samples collected after informed consent was obtained from the patient or companion and Samples that were technically sound (appropriate sample color, adequate sample volume, no leakage, complete demographic information, no sample contamination with other materials and microorganisms).

#### 2.2.1 Appropriate Sample Color

The collected samples met laboratory standards and showed no abnormal color changes. The respiratory samples (nasopharyngeal and oropharyngeal swabs) exhibited no unusual discoloration or contamination with excessive secretions. In this study, all collected samples maintained high-quality color standards, and no changes indicating contamination or sample degradation were reported.

#### 2.2.2 Adequate Sample Volume

The collected samples had sufficient volume for RT-PCR and other relevant tests. The appropriate volume for nasopharyngeal swab samples for RT-PCR was typically between 1 to 3 mL of viral transport medium (VTM).

#### 2.2.3 No Sample Leakage

All samples were collected and transported under standard conditions, and no leakage was reported in the studied samples.

#### 2.2.4 No Sample Contamination

The analyzed samples were free from any contamination with foreign substances or microorganisms and were processed under standard laboratory conditions.

### 2.3 Exclusion criteria

Samples lacking the above information and Patients who withdrew from the study during the study period.

#### 2.3.1 EData Collection and Sampling

Ethical approval for this study was obtained from the Ethics Committee of Semnan University of Medical Sciences (Ethical Code: IR.SEMUMS.REC.1402.055). Nasopharyngeal and oropharyngeal swab samples were collected from patients and sent to the Reference Health Virology Laboratory for molecular diagnosis. Demographic information, past medical history, and clinical details were extracted from electronic medical records.

### 2.4 Viral RNA extraction

Viral RNA was extracted using the Roje kit (Catalog No: SKU- RN983072, ROJE Technologies, Iran) following the manufacturer’s instructions. The procedure began with the initial lysis of the sample using a lysis buffer. This was followed by the addition of ethanol to promote RNA binding to the silica column. To eliminate contaminants, the column was thoroughly washed, and the purified RNA was then eluted and stored at -70°C for subsequent analysis.

### 2.5 Detection of SARS-CoV-2 by real-time RT-PCR assay

RNA was extracted subjected to using COVID-19 One-Step RT-PCR Kit by Real-Time PCR (RT-PCR), and all molecular tests were performed according to the manufacturer’s instructions (COVITECH, Iran). Using the COVITECH kit, RNA was obtained and then submitted to Real-Time Reverse Transcription Polymerase Chain Reaction (RT-PCR). All molecular tests were carried out according to the manufacturer’s instructions (COVITECH, Iran). All the RT-PCR reactions were carried out in the QIAquant 5-Plex 96 Real-Time PCR System (Qiagen, Hilden, Germany).

### 2.6 Bias

To minimize bias, all samples were promptly collected and analyzed under standardized laboratory conditions using reliable, validated methods.

### 2.7 Statistical analysis

All collected data were analyzed using SPSS version 2022 and Stata statistical software. For descriptive analysis, frequency tables, graphs, and measures of central tendency (mean, median, and mode) and dispersion (variance, standard deviation, range, and interquartile range) were employed to characterize the data. To assess associations and test hypotheses, appropriate inferential statistical tests were selected based on the study objectives and the normality of the data distribution. These tests included Pearson’s correlation coefficient, independent t-test, and chi-square test. A significance level of α = 0.05 was used for all statistical tests. This means that a p-value less than 0.05 will be considered statistically significant, indicating that there is sufficient evidence to reject the null hypothesis.

## 3 Results

### 3.1 Patient demographics and clinical characteristics

A total of 86 patients with respiratory symptoms and a positive PCR test for COVID-19 were included in the study. Among these patients, 38 (44.2%) were males and 48 (55.8%) were females. The mean age of the patients was 67.43 years (range: 3-97 years).

**Table 1** summarizes the demographic characteristics of COVID-19 patients included in a study. The data shows a slight majority of females (55.8%) compared to males (44.2%) among the COVID-19 patients. Additionally, the vast majority of patients (91.9%) were married, with only a small percentage (8.1%) being single. Also, the most affected age group was above 61 years old, accounting for 72.1% of the patients. Other age groups had a significantly lower prevalence. It’s important to note that this data only represents a specific patient population studied and may not be generalizable to the entire COVID-19 population.

**Table 1:** Demographic characteristics of COVID-19 patients.

Category	Status	Percentage
**Gender**	Male	44.2%
	Female	55.8%
**Marital Status**	Single	8.1%
	Married	91.9%
**Age Group**	Less than 20 years	2.3%
	21 to 30 years	4.7%
	31 to 40 years	2.3%
	41 to 50 years	4.7%
	51 to 60 years	14.0%
	More than 61 years	72.1%

### 3.2 Comorbidities in severe vs. non-severe patients

A significant proportion of patients presented with pre-existing medical conditions, the most common being hypertension (55.3%), diabetes mellitus (40.7%), and ischemic heart disease (29.2%). **Table 2** provides a detailed comparison of comorbidity prevalence between severe and non-severe cases. It is important to consider that this data only reflects past medical history and may not necessarily indicate active disease at the time of hospitalization. However, the presence of these pre-existing conditions could potentially contribute to disease severity or complications in COVID-19 patients.

**Table 2:** Comparison of past medical history in severe and non-severe COVID-19-positive Patients.

Past medical history	Result	Severe	Non-Severe	P-value
**Diabetes mellitus**	**Positive**	43.1%	37.1%	.740
	**Negative**	56.9%	62.9%	
**Epilepsy**	**Positive**	3.9%	0%	.648
	**Negative**	96.1%	100%	
**Hypertension (HTN)**	**Positive**	51.0%	45.7%	.795
	**Negative**	49.0%	54.3%	
**Hyperlipidemia (HLP)**	**Positive**	21.6%	31.4%	.437
	**Negative**	78.4%	68.6%	
**Cancer**	**Positive**	5.9%	2.9%	.894
	**Negative**	94.1%	97.1%	
**Alzheimer’s**	**Positive**	11.8%	5.7%	.568
	**Negative**	88.2%	94.3%	
**Asthma**	**Positive**	3.9%	11.4%	.362
	**Negative**	94.1%	88.6%	
**Ischemic Heart Disease (IHD)**	**Positive**	19.6%	31.4%	.318
	**Negative**	80.4%	68.6%	
**Chronic Obstructive Pulmonary Disease (COPD)**	**Positive**	7.8%	17.1%	.327
	**Negative**	92.2%	82.9%	
**Cerebrovascular Accident (CVA)**	**Positive**	11.8%	5.7%	.568
	**Negative**	88.2%	94.3%	
**Benign Prostatic Hyperplasia (BPH)**	**Positive**	2.0%	5.7%	.752
	**Negative**	98.0%	94.3%	
**Cirrhosis**	**Positive**	2.0%	0%	1.000
	**Negative**	98.0%	100%	
**Rheumatoid Arthritis (RA)**	**Positive**	7.8%	0%	.240
	**Negative**	92.2%	100%	
**Chronic Kidney Disease (CKD)**	**Positive**	5.9%	5.7%	1.000
	**Negative**	94.1%	94.3%	
**Coronary Artery Disease (CAD)**	**Positive**	0%	2.9%	.849
	**Negative**	100%	97.1%	
**Hypothyroidism**	**Positive**	9.8%	5.7%	.779
	**Negative**	90.2%	94.3%	
**Parkinson’s Disease**	**Positive**	2.0%	5.7%	.738
	**Negative**	98.0%	94.3%	
**End-Stage Renal Disease (ESRD)**	**Positive**	0%	5.7%	.318
	**Negative**	100%	94.3%	
**Heart Failure (HF)**	**Positive**	5.9%	2.9%	.894
	**Negative**	94.1%	97.1%	
**Multiple Sclerosis (MS)**	**Positive**	2.0%	0%	1.000
	**Negative**	98.0%	100%	
**Transient Ischemic Attack (TIA)**	**Positive**	2.0%	0%	1.000
	**Negative**	98.0%	100%	
**Coronary artery bypass grafting (CABG)**	**Positive**	2.0%	2.9%	1.000
	**Negative**	98.0%	97.1%	
**Minor thalassemia**	**Positive**	2.0%	0%	1.000
	**Negative**	98.0%	100%	

### 3.3 RT-PCR CT values and disease severity

59.3% of patients had a CT value categorized as “severe.” Lower CT values generally indicate higher viral loads and 40.7% of patients had a CT value classified as “non-severe.” In addition, CT values below 25 were found to be associated with a higher risk of severe disease, hospitalization, and high viral load in comparison to those patients with CT values higher than 25 (Rabaan et al., 2021; Sadeghi et al., 2022; Legaspi et al., 2023). 31.4% of patients had a SpO2 (oxygen saturation) level below or equal to 90%, indicating potential oxygen desaturation. 68.6% of patients had a SpO2 level above 90%, suggesting normal or less severe oxygenation (**Tables 3**, **4**).

**Table 3:** Treatments Administered to COVID-19 Patients.

Treatment	Used	Not Used
**Remdesivir**	38.7%	61.3%
**Dexamethasone**	46.8%	53.2%
**Acetaminophen**	50%	50%
**Aspirin**	59.3%	40.7%

**Table 4:** Comparison of first SpO2 in severe and non-severe COVID-19-positive patients.

First SpO2	CT		P-value
	Severe	Non-Severe	
**=<90**	37.3%	22.9%	.239
**>90**	62.7%	77.1%	

### 3.4 Laboratory findings and disease severity

**Table 5** summarizes the results of various laboratory tests conducted on COVID-19 patients in the study. The blood sugar levels showed a significant variation, ranging from a minimum of 40 mg/dL to a maximum of 557 mg/dL. The average blood sugar level was 154.05 mg/dL with a standard deviation of 77.249 mg/dL. Lipid profile (TG, LDL, and HDL) levels also exhibited a wide range. Vitamin D levels were relatively low, with a mean of 16.83 and a standard deviation of 1.607. The levels of tests of C-reactive protein (CRP), kidney function (Cr, BUN), liver function (AST, ALT, and ALP), white blood cells (WBC), and hemoglobin (HB) had different ranges as indicated in the table.

**Table 5:** Comparison of laboratory findings in severe and non-severe COVID-19-positive patients.

Laboratory tests	CT	Mean	Std. Deviation	P-value
**FBS**	**Severe**	152.75	83.863	.850
	**Non-Severe**	156.00	67.285	
**TG**	**Severe**	106.38	43.078	.207
	**Non-Severe**	154.27	112.550	
**LDL**	**Severe**	61.82	28.003	.683
	**Non-Severe**	66.45	24.312	
**HDL**	**Severe**	40.33	13.145	.920
	**Non-Severe**	40.90	12.671	
**VitD**	**Severe**	17.50	—	.766
	**Non-Severe**	16.50	2.121	
**CRP**	**Severe**	57.84	41.769	.088
	**Non-Severe**	41.65	40.028	
**Cr**	**Severe**	25.216	12.435	.505
	**Non-Severe**	27.152	13.677	
**AST**	**Severe**	1.203	.663	.091
	**Non-Severe**	1.609	1.235	
**ALT**	**Severe**	45.63	97.689	.533
	**Non-Severe**	64.77	153.197	
**ALP**	**Severe**	51.52	152.420	.742
	**Non-Severe**	64.00	121.941	
**BUN**	**Severe**	206.47	82.976	.322
	**Non-Severe**	183.25	56.288	
**WBC**	**Severe**	10.973	4.571	.350
	**Non-Severe**	9.770	4.998	
**HB**	**Severe**	11.270	2.489	.361
	**Non-Severe**	11.864	2.391	
**Blood pressure**	**Severe**	130.995	22.541	.705
	**Non-Severe**	129.277	17.441	

### 3.5 Correlation between clinical parameters

**Figure 1:**
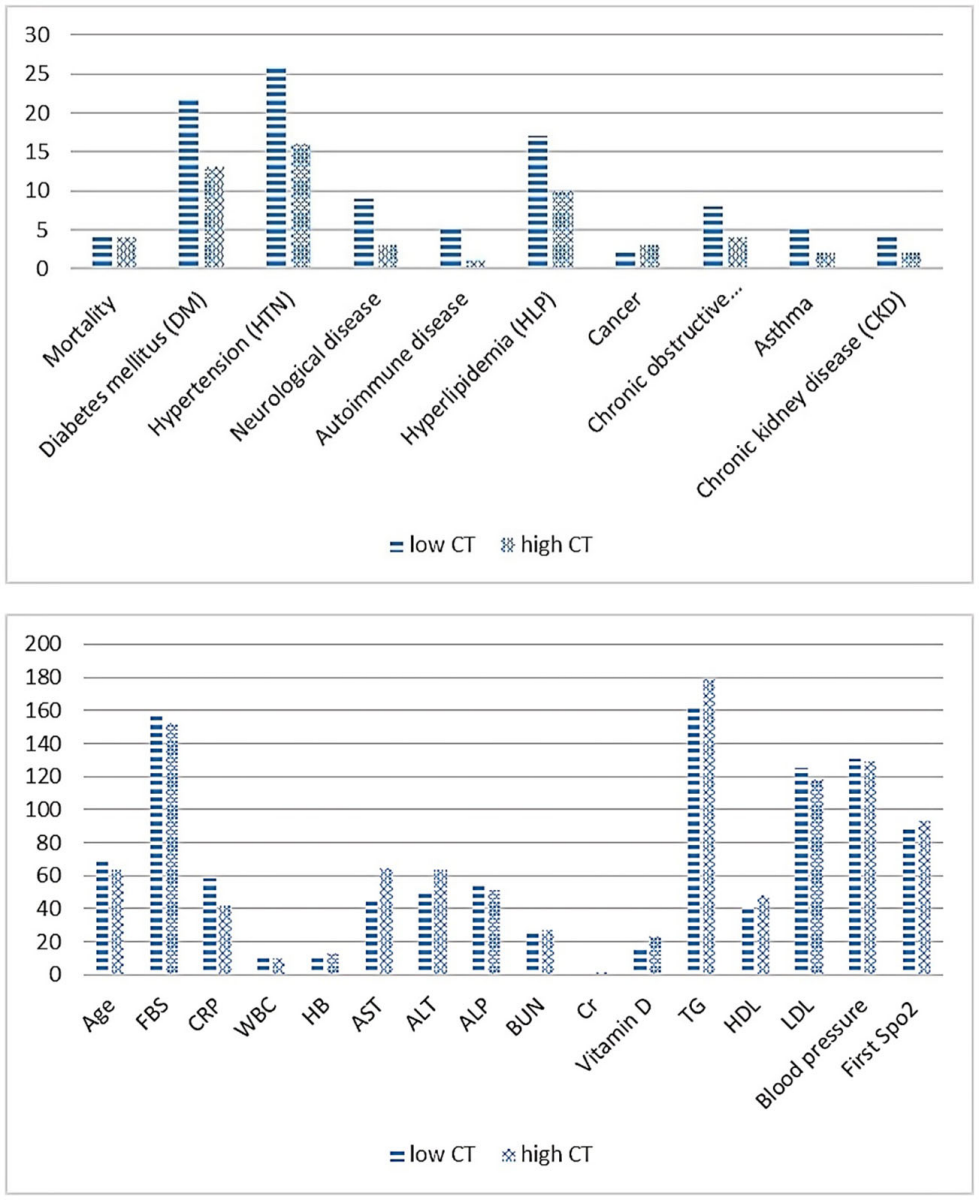
Comparison of comorbidity, laboratory and vital signs findings in patients with severe Vs. non-severe COVID-19.

**Table 6** present’s comorbidity, laboratory, and vital signs findings in hospitalized COVID-19 patients, distinguishing between severe patients (low CT count) and non-severe patients (high CT count). Comorbidity analysis revealed no significant differences between severe and non-severe patients across various conditions, including diabetes mellitus, hypertension, neurological disease, autoimmune disease, hyperlipidemia, cancer, COPD, asthma, age, and chronic kidney disease (Chi-square, p > 0.05) (**Figure 1**).

**Table 6:** Comorbidity, laboratory, and vital signs findings in severe vs. non-severe COVID-19 patients.

Parameters		Severe patients (low CT count)	Non-Severe patients (high CT count)	p-value
**Comorbidities**	**Mortality**	4	4	0.299
	**Diabetes mellitus (DM)**	22	13	0.300
	**Hypertension (HTN)**	26	16	0.068
	**Neurological disease**	9	3	0.233
	**Autoimmune disease**	5	1	0.189
	**Hyperlipidemia (HLP)**	17	10	0.309
	**Cancer**	2	3	0.518
	**Chronic obstructive pulmonary disease (COPD)**	8	4	0.191
	**Asthma**	5	2	0.184
	**Chronic kidney disease (CKD)**	4	2	0.092
	**Age**	69.76	64.03	0.147
**Laboratory tests**	**FBS**	156	152.75	0.044
	**CRP**	57.84	41.65	0.048
	**WBC**	11.97	9.77	0.035
	**HB**	11.27	12.86	0.045
	**AST**	45.63	64.77	0.322
	**ALT**	51.52	64.00	0.111
	**ALP**	53.55	51.35	0.322
	**BUN**	25.21	27.15	0.446
	**Cr**	1.60	1.20	0.020
	**Vitamin D**	17.80	23.21	0.092
	**TG**	161.25	178.60	0.290
	**HDL**	41.88	48.40	0.056
	**LDL**	125.11	118.20	0.522
**Vital sings**	**Blood pressure**	130.99	129.2	0.129
	**First Spo2**	89.88	93.14	0.011

Chi-square and independent samples t-test analysis was conducted. The significance level was set at 0.05.

In laboratory tests, significant differences were observed in fasting blood sugar (FBS), CRP, WBC, hemoglobin (HB), and creatinine (Cr) levels between the two groups (independent samples t, p ≤ 0.05). Regarding vital signs, there was a significant difference in the first SpO2 measurement between severe and non-severe patients, with the former showing lower oxygen saturation levels (independent samples t, p ≤ 0.05).

**Table 7** illustrates the correlation among various clinical parameters in COVID-19 patients. Age demonstrates a weak positive correlation with BUN (Blood Urea Nitrogen) (r = 0.283, p = 0.009) and a weak negative correlation with SPo2 (oxygen saturation) (r = -0.315, p = 0.003). This suggests that as patient’s age, their BUN levels tend to increase, and they also exhibit slightly lower oxygen saturation levels. Additionally, FBS exhibits a significant negative correlation with Vitamin D (r = -0.999, p = 0.020), indicating an association between elevated FBS levels and decreased Vitamin D levels. Furthermore, there exists a positive correlation between FBS and TG levels (r = 0.467, p = 0.021), signifying elevated FBS levels’ association with increased TG levels in COVID-19 patients.

**Table 7:** Correlation between clinical parameters in COVID-19 patients.

Clinical Parameters		Age	FBS	CRP	WBC	HB	AST	ALT	ALP	BUN	Cr	Vit D	TG	HDL	LDL	SPo2
Age	Correlation	1.00	0.178	0.215	0.115	0.137	0.059	0.051	-0.025	0.283*	0.134	-0.288	-0.021	-0.075	-0.070	-0.315*
	P value	0	0.103	0.52	0.394	0.302	0.636	0.705	0.866	0.009	0.225	0.814	0.923	0.740	0.758	0.003
FBS	Correlation	0.178	1.00	-0.127	0.159	0.110	-0.115	-0.078	0.026	0.271*	0.203	-0.999*	0.467*	-0.213	-0.062	-0.053
	P value	0.103	0	0.257	0.238	0.407	0.352	0.564	0.866	0.013	0.064	0.020	0.021	0.340	0.874	0.631
CRP	Correlation	0.215	-0.127	1.00	0.261	-0.016	0.322*	0.311	0.261	0.064	0.421	-0. 210	-0.305	-0.072	0.116	-0.306*
	P value	0.52	0.257	0	0.172	.092	0.008	0.235	0.625	0.263	0.130	0.088	0.147	0.749	0.606	0.005
WBC	Correlation	0.115	0.159	0.261	1.00	-0.023	0.289	-0.007	0.090	0.279	0.052	-0.072	-0.203	0.120	0.265	-0.625
	P value	0.394	0.238	0.172	0	0.611	0.171	0.976	0.723	0.036	0.774	0.746	0.419	0.230	0.288	0.760
HB	Correlation	0.137	0.110	-0.016	-0.023	1.00	0.253	0.552	0.420	-0.566	0.237	0.116	0.090	0.026	-0.592*	0.080
	P value	0.302	0.407	.092	0.611	0	0.068	0.128	0.080	0.153	0.328	0.606	0.723	0.066	0.016	0.060
AST	Correlation	0.059	-0.115	0.322*	0.289	0.253	1.00	-0.227	-0.592	0.400	-0.211	0.305	0.030	-0.475*	-0.211	-0.020
	P value	0.636	0.352	0.008	0.171	0.068	0	0.310	0.160	0.053	0.386	0.147	0.902	0.040	0.386	0.213
ALT	Correlation	0.051	-0.078	0.311	-0.007	0.552	-0.227	1.00	0.009	-0.149	0.030	0.184	0.096	0.092	-0.242	0.420
	P value	0.705	0.564	0.235	0.976	0.128	0.310	0	0.974	0.509	0.902	0.436	0.703	0.122	0.350	0.100
ALP	Correlation	-0.025	0.026	0.261	0.090	0.420	-0.592	0.009	1.00	-0.283	0.849	-0.387	-0.034	-0.310	0.815*	0.010
	P value	0.866	0.866	0.625	0.723	0.080	0.160	0.974	0	0.202	0355	0.083	0.905	0.250	0.001	0.090
BUN	Correlation	0.283*	0.271*	0.064	0.279	-0.566	0.400	-0.149	-0.283	1.00	0.777*	0.233	0.289	-0.071	-0.066	0.050
	P value	0.009	0.013	0.263	0.036	0.153	0.053	0.509	0.202	0	0.001	0.850	0.181	0.565	0.259	0.820
Cr	Correlation	0.134	0.203	0.421	0.052	0.237	-0.211	0.030	0.849	0.777*	1.00	-0.203	0.400	-0.011	-0.149	0.096
	P value	0.225	0.064	0.130	0.774	0.328	0.386	0.902	0355	0.001	0	0.419	0.053	0.055	.509	0.140
Vit D	Correlation	-0.288	-0.999*	-0. 210	-0.072	0.116	0.305	0.184	-0.387	0.233	-0.203	1.00	-0.020	0.078	0.500	0.266
	P value	0.814	0.020	0.088	0.746	0.606	0.147	0.436	0.083	0.850	0.419	0	0.090	0.082	0.922	0.640
TG	Correlation	-0.021	0.467*	-0.305	-0.203	0.090	0.030	0.096	-0.034	0.289	0.400	-0.020	1.00	-0.387	-0.092	-0.137
	P value	0.923	0.021	0.147	0.419	0.723	0.902	0.703	0.905	0.181	0.053	0.090	0	0.83	0.684	0.524
HDL	Correlation	-0.075	-0.213	-0.072	0.120	0.026	-0.475*	0.092	-0.310	-0.071	-0.011	0.078	-0.387	1.00	-0.387	0.266
	P value	0.740	0.340	0.749	0.230	0.066	0.040	0.122	0.250	0.565	0.055	0.082	0.83	0	0.83	0.455
LDL	Correlation	-0.070	-0.062	0.116	0.265	-0.592*	-0.211	-0.242	0.815*	-0.066	-0.149	0.500	-0.092	0.184	1.00	-0.011
	P value	0.758	0.874	0.606	0.288	0.016	0.386	0.350	0.001	0.259	.509	0.922	0.684	0.436	0	0.962
SPo2	Correlation	-0.315*	-0.053	-0.306*	-0.625	0.080	-0.020	0.420	0.010	0.050	0.096	0.266	-0.137	0.266	-0.011	1.00
	P value	0.003	0.631	0.005	0.760	0.060	0.213	0.100	0.090	0.820	0.140	0.640	0.524	0.455	0.962	0

*Pearson’s correlation analysis was performed, and statistically significant correlations (p < 0.05) are indicated by bold numbers.

The correlation coefficient between FBS and BUN (r = 0.271, p = 0.013) suggests a moderate positive correlation between fasting blood sugar and blood urea nitrogen levels. This indicates that as FBS levels rise, there is a tendency for BUN levels to increase as well. Additionally, there is a moderate positive correlation between CRP and AST (Aspartate Aminotransferase) (r = 0.322, p = 0.008), indicating that elevated CRP, a marker of inflammation, is associated with slightly higher AST levels, an enzyme found in the liver.

Furthermore, the results reveal a moderate negative correlation between CRP and SPo2 (r = -0.306, p = 0.005). Additionally, HB (Hemoglobin) exhibits a moderate negative correlation with LDL (Low-Density Lipoprotein) (r = -0.592, p = 0.016), suggesting a relationship between lower hemoglobin levels and increased LDL levels. Also, there was a strong positive correlation between ALP (alkaline phosphatase) and LDL.

Moreover, a weak positive correlation is observed between BUN and Creatinine (Cr) (r = 0.777, p = 0.001), both markers of kidney function. Lastly, there is a moderate negative correlation between AST and HDL (r = -0.475, p = 0.040), indicating a connection between elevated AST levels and decreased HDL levels. These correlations highlight the complex interplay between various physiological processes and biochemical parameters during COVID-19 infection, reflecting the systemic impact of the disease on multiple organ systems.

## 4 Discussion

The emergence of the COVID-19 pandemic caused by the SARS-CoV-2 virus has prompted global efforts in clinical diagnostics to facilitate the timely and accurate identification of cases. Various testing methods, including real-time reverse transcription-PCR (RT-PCR), are employed in clinical laboratories to diagnose and monitor COVID-19. The Cycle Threshold in RT-PCR serves as a crucial criterion, correlating lower values with higher virus loads and potentially severe disease outcomes (Tomo et al., 2020). The analysis revealed that most hospitalized patients were elderly, with an average age of 67.43 years, and frequently had pre-existing conditions like hypertension (55.3%) and diabetes mellitus (40.7%). Although no significant differences in comorbidities were identified between patients with high and low Ct values, the overall prevalence of chronic illnesses among hospitalized individuals is consistent with earlier findings highlighting how these conditions worsen COVID-19 outcomes. Research indicates that advanced age and comorbidities heighten disease severity due to compromised immune responses and a greater vulnerability to systemic inflammation (Alkhouli et al., 2020; Jin et al., 2020; Mukherjee and Pahan, 2021).

A considerable portion of patients in our study presented with pre-existing chronic health conditions, notably hypertension (55.3%), diabetes mellitus (40.7%), and ischemic heart disease (29.2%). However, no significant differences were observed in the prevalence of these comorbidities between patients with low and high CT counts in RT-PCR. Similarly, Liu et al. (2020) reported higher prevalence rates of diabetes, coronary artery disease/cardiovascular disease (CAD/CVD), and hypertension compared to chronic pulmonary disease. However, their findings did not align with ours in terms of the association between comorbidities and CT counts in RT-PCR (Liu et al., 2020). Moreover, Sanyaolu et al. (2020) highlighted the increased severity and progression of COVID-19 in patients with comorbidities such as hypertension or diabetes mellitus, particularly among older individuals aged 65 and above. These patients are more prone to ICU admission and mortality from COVID-19 (Sanyaolu et al., 2020).

In the present study, COVID-19 patients with higher viral loads in their RT-PCR tests (or low CT counts, indicating severity) exhibited significantly higher average FBS levels compared to those with lower viral loads. In the study of Shahcheraghi et al. (2022), no significant difference was observed for D-Dimer, BS, and FBS based on 4 factors including gender, age, hospitalization, and recovery (Shahcheraghi et al., 2022). The increase in sugar levels in COVID-19 patients may be attributed to factors such as insulin resistance, hyperglycemia due to inflammation, prolonged bed rest, disease-related stress, elevated inflammatory cytokines, and CRP levels, and the effects of medications used in COVID-19 treatment. This phenomenon may also impact systemic inflammatory responses and immune system function (Zahedi et al., 2023).

Inflammatory biomarkers, such as CRP and WBC, showed significant elevation in patients with lower CT values. This aligns with earlier research indicating that higher viral loads are associated with heightened inflammation, likely driven by excessive immune activation. The cytokine storm, marked by increased levels of IL-6, TNF, and IL-10, has been implicated in severe COVID-19 cases and is closely linked to multi-organ failure. Moreover, hemoglobin levels were considerably lower in patients with severe disease, pointing to the potential impact of inflammation-induced suppression of erythropoiesis. Previous studies have highlighted the connection between reduced hemoglobin and poor COVID-19 outcomes, identifying anemia as a factor associated with higher mortality risk. Markers of renal function, such as creatinine and BUN, were notably elevated in patients with severe disease and lower CT values. This indicates that viral load could play a role in kidney injury, possibly through direct viral invasion of renal tissues or as a consequence of systemic inflammation and hypoxia. Acute kidney injury (AKI) has been commonly observed in critically illness COVID-19 patients, with research showing that impaired renal function is a strong predictor of mortality in severe cases (Zhong et al., 2021; Chang et al., 2022). Overall, these findings suggest a complex interplay between metabolic parameters, inflammation, and immune activation in COVID-19 patients, highlighting the importance of monitoring and managing metabolic and inflammatory markers in clinical practice.

An important observation highlighted in this study is the relationship between CT values and SpO2 levels. Patients with lower CT values showed markedly reduced oxygen saturation, demonstrating the effect of viral replication on respiratory function. This underscores the influence of viral load on disease severity, as elevated rates of viral replication can contribute to greater lung inflammation and alveolar injury, ultimately leading to hypoxemia (Kalani et al., 2023; Moghadaci et al., 2024). Although strong associations were observed between CT values and markers such as inflammation, renal function, and oxygenation status, no significant correlations emerged with other laboratory parameters, including ALT, AST, ALP, vitamin D, TG, HDL, and LDL. These findings indicate that while viral load plays a pivotal role in determining disease severity, other factors like immune response, metabolic health, and genetic predispositions may also shape patient outcomes (Krishnan et al., 2022; Adamidis et al., 2024).

These correlations highlight the complex interplay between various physiological processes and biochemical parameters during COVID-19 infection, reflecting the systemic impact of the disease on multiple organ systems. The following are some of the study’s limitations: Firstly, the current study has a rather tiny sample size. Second, certain information was not recorded, which made it impossible for us to get precise and comprehensive clinical data on every patient. Furthermore, the absence of some data could have an impact on the study’s findings.

## 5 Conclusion

The correlations observed in COVID-19 patients highlight the intricate interplay between physiological processes and biochemical parameters, indicating the systemic impact of the disease on multiple organ systems. Lower CT values, potentially indicating higher viral load, seem to be associated with various physiological changes, including higher fasting blood sugar levels, Increased inflammation (CRP and WBC), higher hemoglobin levels, kidney dysfunction (Increased Cr and BUN), and lower initial SpO2 in patients. These findings could potentially aid in risk stratification and clinical decision-making for COVID-19 patients. However, further research and clinical validation are necessary to confirm these associations and their implications for patient management.

## Data Availability

The datasets presented in this study can be found in online repositories. The names of the repository/repositories and accession number(s) can be found in the article/supplementary material.
